# Recent Progress in Flexible Wearable Sensors for Real-Time Health Monitoring: Materials, Devices, and System Integration

**DOI:** 10.3390/mi16101124

**Published:** 2025-09-30

**Authors:** Jianqun Cheng, Ning Xue, Wenyi Zhou, Boqi Qin, Bocang Qiu, Gang Fang, Xuguang Sun

**Affiliations:** School of Electronics and Communication Engineering, Quanzhou University of Information Engineering, Quanzhou 362000, China; jcheng@qzuie.edu.cn (J.C.); xuening@qzuie.edu.cn (N.X.);

**Keywords:** flexible sensors, wearable devices, health monitoring

## Abstract

Flexible and wearable sensors have emerged as transformative technologies in the field of real-time health monitoring, offering non-invasive, continuous, and personalized healthcare solutions. These devices are designed to conform intimately to the human body, enabling seamless detection of vital physiological and biochemical signals under dynamic conditions. Recent advancements in material science and device engineering have led to the development of sensors with enhanced sensitivity, biocompatibility, and wearability, addressing the growing demand for preventive healthcare and remote patient monitoring. This review provides a comprehensive overview of the progress in flexible wearable sensors, including novel materials, sensor designs, and system integration strategies. It begins by surveying the latest advances in substrate and functional materials and hybrid structures that enable mechanical flexibility, skin conformability, and high sensitivity. The review then examines various sensor mechanisms and their implementation in monitoring vital signs, physical activity, and chronic diseases. Real-world applications are explored in depth, covering scenarios from cardiovascular and respiratory monitoring to motion tracking and rehabilitation support. Despite the significant strides made, challenges related to material robustness, sensor accuracy, and multi-modal integration remain, and this review discusses these challenges alongside potential future directions for enhancing the functionality and adoption of flexible wearable sensor systems.

## 1. Introduction

In recent years, the convergence of flexible electronics, advanced materials science, and biomedical engineering has catalyzed the rapid development of wearable health monitoring systems. These systems are designed to continuously and non-invasively track physiological signals such as heart rate, respiration, body temperature, blood pressure, and biochemical markers, offering unprecedented opportunities for real-time and personalized healthcare. With the growing global emphasis on preventive medicine and remote patient care, flexible wearable sensors have emerged as a cornerstone technology in digital health, enabling continuous monitoring outside traditional clinical environments [1,2].

Unlike conventional rigid electronics, flexible and stretchable sensors are capable of conforming intimately to the dynamic surfaces of human skin or internal tissues. This mechanical adaptability, combined with advances in lightweight and biocompatible materials, makes them ideal for long-term, unobtrusive wear [3]. In addition, as health monitoring technologies are evolving from intermittent centralized diagnostic paradigms toward real-time, personalized and decentralized systems, sensing technologies need to make necessitates significant advancements in the real-time performance, flexibility and wearability [4,5,6].

**Figure 1:**
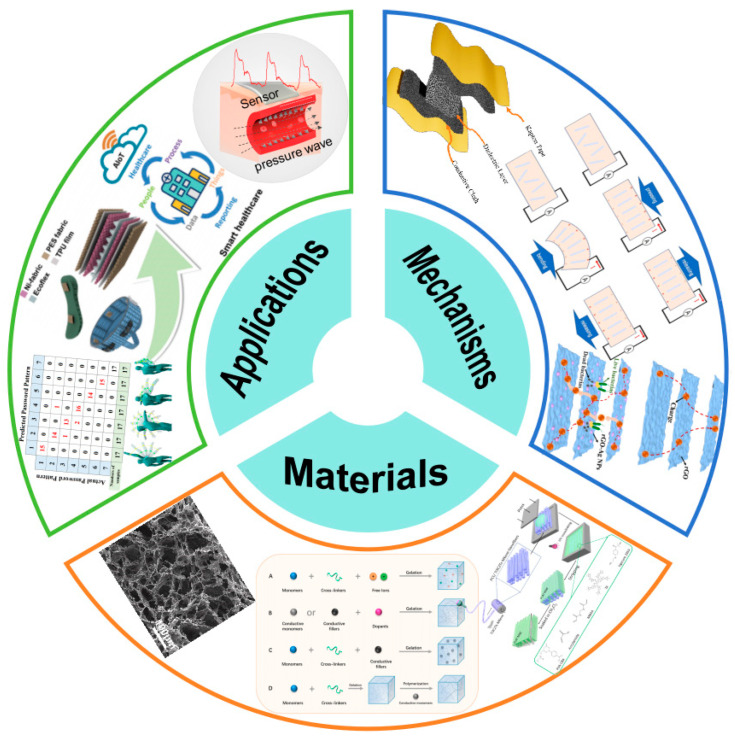
Multi-level innovations of flexible wearable sensors promote the development of real-time health monitoring. Reprinted/adapted with permission from [29]. 2021, Elsevier Publishing Ltd. Reprinted/adapted with permission from [30]. 2021, Elsevier Publishing Ltd. Reprinted/adapted with permission from [31]. 2023, American Chemical Society. Reprinted/adapted with permission from [13]. 2021, John Wiley and Sons Publishing Ltd. Reprinted/adapted with permission from [32]. 2021, John Wiley and Sons Publishing Ltd. Reprinted/adapted with permission from [33]. 2022, Elsevier Publishing Ltd. Reprinted/adapted with permission from [34]. 2023, Elsevier Publishing Ltd.

Real-time monitoring is critical for capturing dynamic physiological signals and providing timely feedback. Only sensors with high temporal resolution and continuous data acquisition capabilities can deliver accurate diagnostics, early warnings of abnormal health conditions, and reliable assessments of therapeutic outcomes. Flexibility, at the material and structural levels, enables sensors to conform to the complex curvatures of the human body without compromising performance [7,8,9]. Flexible electronics ensure mechanical compliance under skin deformation and motion, thus enhancing comfort and minimizing the risk of irritation or injury commonly associated with rigid devices. Furthermore, flexible materials often offer improved biocompatibility and long-term stability in epidermal or implantable applications. Wearability serves as the essential link between sensing capability and practical usability. Wearable health monitoring systems must be lightweight, low-power, and capable of wireless communication [10,11,12]. These features allow seamless integration into daily life through skin-attachable patches, smart textiles, contact lenses, or even implantable platforms, enabling continuous, unobtrusive monitoring in both clinical and non-clinical settings. As shown in Figure 1, the advancement of health monitoring systems hinges on innovations across multiple domains, including sensitive materials, micro/nano device engineering, and system-level integration. A truly effective platform must embody the synergy of real-time sensing, mechanical flexibility, and wearable design, while also leveraging interdisciplinary approaches from materials science, biomedical engineering, electronics or artificial intelligence [13,14,15,16,17].

The rapid advancement of flexible and wearable sensors for real-time health monitoring is fundamentally driven by interdisciplinary innovations. These technological developments are not isolated and they represent the convergence of progress across materials science, micro/nano fabrication, integrated electronics and artificial intelligence [18]. Each domain contributes critical enabling factors that collectively propel the evolution of wearable healthcare systems toward higher functionality, reliability, and user adaptability. First, advanced functional materials serve as the foundation of flexible sensors by offering exceptional properties such as mechanical stretchability, skin conformability, biocompatibility, and high sensitivity to physiological stimuli. Materials including stretchable polymers, conductive hydrogels, graphene, and other two-dimensional nanomaterials have enabled the fabrication of sensors that can closely interface with the human body for accurate and long-term biosignal acquisition [19,20].

Second, the advancement of sensor device architectures and operating principles has significantly contributed to the evolution of real-time health monitoring systems. High-performance sensors with optimized transduction mechanisms such as piezoresistive, capacitive, piezoelectric, and triboelectric effects have enabled precise and rapid conversion of various physiological signals into readable electrical outputs. These mechanisms offer advantages such as fast response, low power consumption, and high sensitivity, making them well-suited for detecting subtle biological changes, including pulse pressure variations, skin temperature shifts, and muscle movements. In particular, the use of multi-modal sensing strategies has expanded the functional capabilities of wearable platforms [21,22].

Real-time health monitoring applications not only demonstrate the functional versatility of emerging wearable systems but also underscore their transformative potential in preventive, diagnostic, and therapeutic healthcare. Wearable platforms incorporating electrocardiography (ECG) and temperature sensors offer non-invasive, continuous tracking of these indicators with accuracy levels within ±6 bpm for ECG heart rate detection and standard deviation 0.14% for temperature monitoring, approaching clinical reference devices [23,24]. Wearable sensors have shown great promise in motion and activity tracking, which is essential for gait analysis, posture classification and rehabilitation assessment [25,26,27,28]. The management of chronic diseases and long-term rehabilitation has greatly benefited from wearable sensor integration. These applications contribute to personalized treatment and remote healthcare by reducing the dependence on hospital-based diagnostics. This review aims to provide a comprehensive overview of recent progress in the field of flexible wearable sensors for real-time health monitoring. We discuss the development of novel materials, the design of high-performance sensor devices, and strategies for system-level application. In addition, we highlight current challenges and future directions that are crucial for transforming flexible sensors into clinically reliable, user-friendly, and commercially viable health monitoring platforms.

## 2. Materials for Flexible and Wearable Sensors

The development of flexible and wearable sensors for real-time health monitoring relies fundamentally on the selection and engineering of materials that can meet the unique demands of mechanical compliance, biocompatibility, and long-term stability. In contrast to conventional rigid electronics, wearable systems must conform intimately to complex, dynamic human surfaces such as skin or internal tissue, often under continuous deformation due to motion, perspiration, and environmental exposure. Therefore, the choice of both structural substrates and functional active materials plays a crucial role in determining device performance, durability, and user comfort [35,36,37].

Recent progress in materials science has introduced a diverse range of substrates that are thin, lightweight, stretchable, and breathable, enabling seamless integration with the human body. Concurrently, the incorporation of advanced functional materials has expanded the sensing capabilities to encompass a wide spectrum of physiological and biochemical parameters. These include electrical conductivity, mechanical sensitivity, thermal response, and chemical selectivity, which are essential for accurately capturing complex biological signals.

### 2.1. Substrate Materials

**Figure 2:**
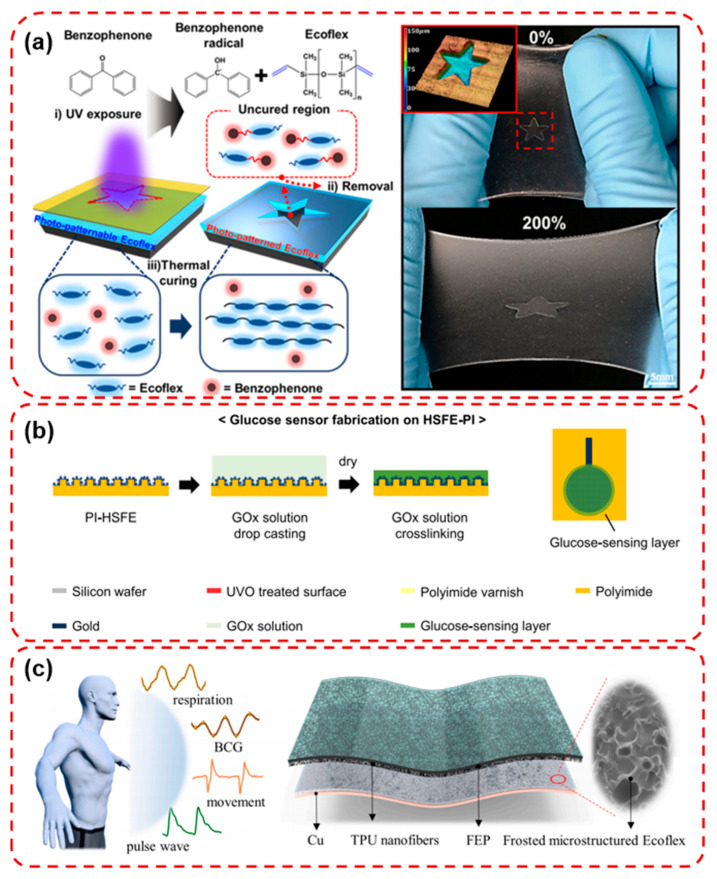
(**a**) Schematic illustration of photopatterning process using photo-patternable Ecoflex (PPE) and photograph of photo-patterned Ecoflex as a star shape on Ecoflex substrate when stretched up to 200% [43]. (**b**) A glucose sensor is fabricated by immobilizing glucose oxidase (GOx) on HSFE-PI. The immobilization process is performed by crosslinking glutaraldehyde (GLA) and bovine serum albumin (BSA) [44]. (**c**) Different kinds of human physiological signals include respiration, BCG, movement, and pulse wave and the schematic structure diagram of the single sensor [45].

The substrate serves as the mechanical backbone of flexible and wearable sensors, providing physical support for active sensing components while enabling intimate contact with soft and dynamic human tissues. An ideal substrate must exhibit a combination of flexibility, stretchability, biocompatibility, and environmental stability, without compromising the performance of the integrated functional layers. Commonly used substrate materials include polyimide (PI), polydimethylsiloxane (PDMS), Ecoflex, hydrogels, textiles, and paper-based substrates [38,39,40,41]. Among them, PI offers excellent thermal stability and moderate flexibility, making it suitable for microscale patterning and device integration [29,30,42]. PDMS and Ecoflex are elastomeric polymers with superior stretchability and skin conformity, widely used in epidermal electronics and wearable strain sensors. Hydrogels, due to their high-water content and tissue-like mechanical properties, are particularly attractive for bio-interfacing and transdermal sensing applications. Textiles and paper-based substrates provide low-cost, breathable, and disposable platforms, facilitating large-area fabrication and consumer-level applications. Kim et al. developed an intrinsically stretchable multi-biochemical sweat sensor by introducing photo-patternable Ecoflex as a process-compatible encapsulation layer, enabling precise patterning and improved strain tolerance, as shown in Figure 2a. The Ecoflex encapsulation effectively dissipated strain energy, yielding 50% higher conductance under 250% strain and maintaining stability over 1000 stretching cycles. By integrating Au nanoparticle-modified Ag electrodes, the device achieved enhanced biocompatibility and electrochemical performance, allowing simultaneous, interference-free monitoring of glucose, lactate, pH, and humidity in sweat. On-body tests demonstrated reliable, real-time sweat analysis that closely matched commercial assay kits, underscoring its potential for continuous, non-invasive health monitoring [43].

In addition to mechanical properties, biocompatibility and gas permeability are critical for long-term wearability. For example, PDMS and hydrogels exhibit high oxygen permeability, which minimizes skin irritation and improves user comfort. Meanwhile, recent advances in surface engineering and material composites have enabled tunable porosity and moisture management, further enhancing skin integration. The choice of substrate not only affects the mechanical behavior of the device but also influences signal fidelity, especially in dynamic conditions such as joint movement or perspiration. Therefore, the integration of substrates with tailored mechanical and biochemical properties is essential for building reliable, skin-conformable sensors capable of continuous, real-time physiological monitoring. In Figure 2b, Jeung et al. developed hierarchically structured flexible electrodes on polyimide (HSFE-PI) by combining a micro-casting process with gold nanoparticle electrodeposition, achieving a 2.06-fold increase in effective surface area compared to planar electrodes. The electrodes exhibited excellent mechanical reliability under 10,000 bending cycles and significantly improved biosignal acquisition, enhancing EMG signal-to-noise ratio by 2.5 times. Moreover, HSFE-PI-based glucose sensors demonstrated 1.42-fold higher sensitivity than conventional counterparts, highlighting the dual applicability for electrophysiological and biochemical monitoring [44]. Wang et al. developed a triboelectric-based 3 × 1 flexible pressure sensor array using frosted microstructured Ecoflex films and electrospun TPU nanofibers, enabling epidermal pulse wave monitoring at multiple radial artery positions, as shown in Figure 2c. The single small-area sensor (6 × 6 mm^2^) exhibited high sensitivity (0.14 V/kPa), fast response time (22 ms), stable operation over 7000 cycles, and effective detection of subtle physiological signals such as respiration, ballistocardiogram, and heartbeat. Integrated into a wristband system, the sensor array simultaneously captured pulse waves, allowing extraction of key cardiovascular parameters including pulse transit time (PTT) and pulse wave velocity (PWV) [45].

### 2.2. Functional Materials

Functional materials play a central role in determining the sensitivity, selectivity, and signal transduction mechanisms of flexible and wearable sensors. These materials are typically integrated as the active layer that interacts directly with physiological stimuli such as pressure, temperature, strain, or biochemical species and convert them into measurable electrical signals [46,47,48]. The ideal functional material should exhibit high electrical conductivity or sensitivity, mechanical compliance, environmental stability, and compatibility with low-temperature, solution-based fabrication processes suitable for flexible substrates [49,50,51,52].

**Figure 3:**
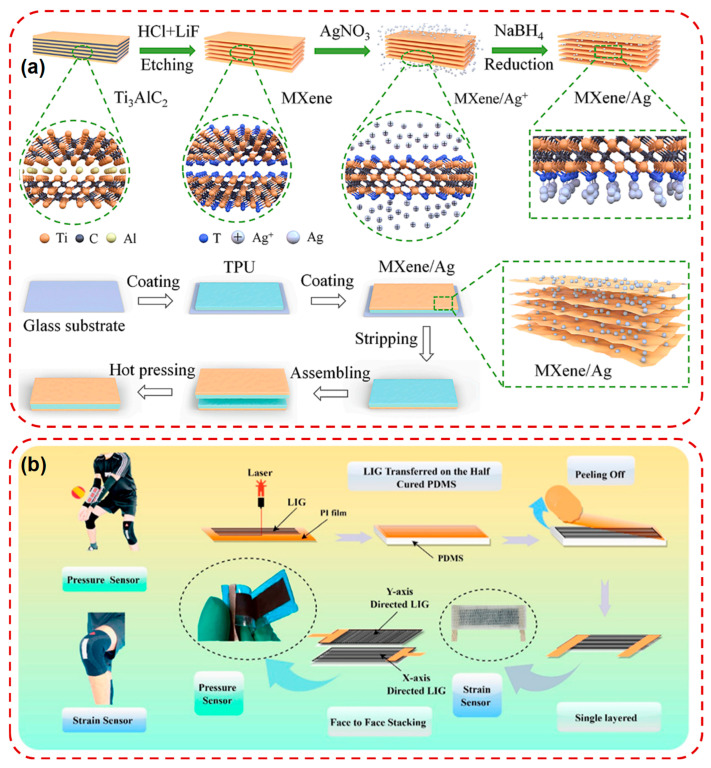
(**a**) Schematic diagram of the preparation procedures of MXene/Ag heterostructure electrodes and the schematic illustration of the fabrication process of the piezoionic sensor based on MXene/Ag electrode [56]. (**b**) Schematics of the fabrication process of the LIG pattern via laser-induced pyrolysis technology and design strain and a pressure sensor for the volleyball sports garment [57]. Reprinted/adapted with permission from [56]. 2024, Elsevier Publishing Ltd. Reprinted/adapted with permission from [57]. 2022, American Chemical Society Publishing Ltd.

A diverse array of material classes has been explored for sensor functionality. Conductive polymers such as poly(3,4-ethylenedioxythiophene):poly(styrenesulfonate) (PEDOT:PSS), offer tunable electrical and mechanical properties, along with excellent film-forming ability [23,53]. These materials are frequently used in strain, temperature, and electrochemical sensors. Carbon-based nanomaterials, including graphene and carbon nanotubes (CNTs) provide exceptional electrical conductivity, high surface area, and chemical stability. Their intrinsic flexibility and functionalizability make them ideal candidates for multimodal sensing platforms, such as electrophysiological recording and biochemical detection. MXenes a class of two-dimensional transition metal carbides and nitrides, have emerged as promising materials due to their metallic conductivity, hydrophilicity, and processability into flexible films. Their use has been reported in pressure, humidity, and chemical sensors, often with superior sensitivity and fast response time [31,54]. What’s more, metal nanowires particularly silver nanowires (AgNWs), are employed as transparent and stretchable conductors in wearable devices [55]. Their percolative networks maintain conductivity under deformation, making them suitable for capacitive and resistive-type sensors. Nanoparticles, piezoelectric, and thermoelectric materials are also widely applied in self-powered sensors, where mechanical or thermal energy is directly converted into electrical output without the need for external power sources. Li et al. developed a self-powered piezoionic sensor based on MXene/Ag nanoparticle heterostructure electrodes, addressing the low output voltage and poor stability of conventional ionic sensors as shown in Figure 3a. The interlayer insertion of Ag nanoparticles expanded MXene spacing, enhanced ion storage and transport, and enabled a stable voltage output of 11.1 mV under 0.7% strain with 95% retention over 13,000 s of cyclic bending. The device demonstrated reliable detection of diverse human motions, real-time evaluation of CPR compression quality, and even information transmission via Morse code encoding [56]. Raza et al. introduced a multifunctional wearable sensor based on laser-induced graphene (LIG) laminated onto PDMS and integrated into elastic sports fabrics for real-time volleyball monitoring as shown in Figure 3b. By configuring single-layer LIG/PDMS for strain sensing and stacked orthogonal layers for pressure sensing, the induced graphene textile device achieved high linearity, fast response, and durability over wide strain (0–100%) and pressure (0–220 kPa) ranges. The smart sportswear enabled diverse applications including player position tracking, spike force measurement, reception detection, and foul recognition, providing coaches with actionable training insights [57].

The integration of these functional materials with soft substrates enables the development of highly conformable sensors capable of detecting subtle physiological signals. Moreover, the synergistic use of multiple materials such as nanocomposites combining CNTs and polymers or hybrid structures with MXene and metal nanowires allows for enhanced sensing performance through multi-mechanism interactions such as piezoresistive, capacitive, or thermoelectric effects. As the field progresses, continued innovation in material synthesis, doping, and surface functionalization will be key to achieving higher sensitivity, broader detection range, and environmental robustness in real-world healthcare settings.

### 2.3. Biocompatibility and Long-Term Stability

For wearable sensors to be deployed reliably in continuous and real-time health monitoring applications, biocompatibility and long-term operational stability are of paramount importance. Unlike traditional rigid electronics, flexible and skin-mounted devices must maintain intimate contact with human skin over extended periods without inducing irritation, allergic reactions, or mechanical discomfort. Biocompatibility refers not only to the inertness of the material with respect to biological systems, but also to its ability to support safe and effective physiological interactions. Materials such as hydrogels, Ecoflex, PDMS, and silk fibroin have been extensively studied for their skin-conformal properties, moisture permeability, and low cytotoxicity. Natural and synthetic materials are often subjected to surface functionalization to enhance adhesion, prevent biofouling, and reduce inflammatory responses [58,59,60,61].

**Figure 4:**
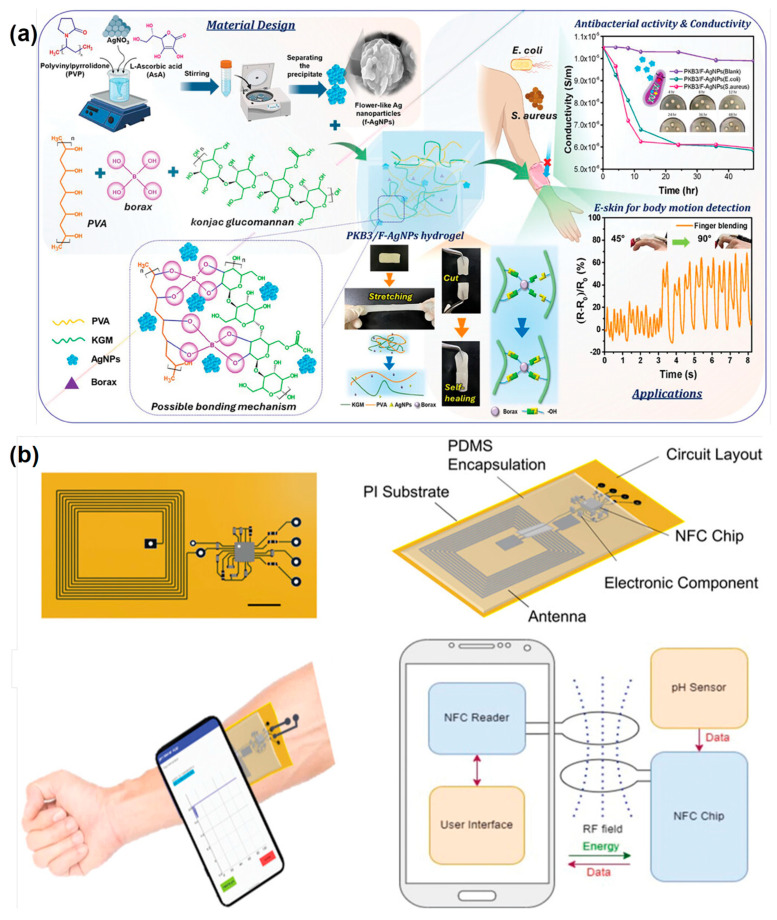
(**a**) Proposed conceptual illustration of multifunctional PKB3/F-AgNPs hydrogels [62]. (**b**) Top view of NFC-based electronic/communication circuitry module (scale bar: 1 cm). The NFC-based electronic/communication circuitry module is encapsulated in PDMS. Wireless power and data exchange between the WB2F3D sensor system and a phone in close proximity. A block diagram showing the concept of wireless power and data exchange between the WB2F3D sensor system and a smartphone, enabling on-demand, battery-free, and real-time pH sensing. The real-time measured data will be displayed on the smartphone app [63]. Reprinted/adapted with permission from [62]. 2024, John Wiley and Sons Publishing Ltd. Reprinted/adapted with permission from [63]. 2023, John Wiley and Sons Publishing Ltd.

At the same time, long-term stability requires that the materials and sensor interfaces remain chemically, mechanically and electrically stable under dynamic environmental and usage conditions, such as repeated skin deformation, exposure to sweat, oils and UV radiation. Encapsulation layers use breathable but protective films to shield the active components from moisture and oxidation, surface coatings with hydrophobic or anti-corrosive properties to resist degradation caused by biological fluid, and self-healing materials that can recover from microcracks and damage, prolonging device lifetime. As shown in Figure 4a, Cheng et al. developed a hierarchically structured antibacterial and biocompatible hydrogel (PKB/F-AgNPs) by integrating polyvinyl alcohol, konjac glucomannan, borax, and flower-like silver nanoparticles, achieving high stretchability (1041%), robust self-healing (83%), and superior cell viability (128%). The incorporation of flower-shaped AgNPs endowed the hydrogel with strong antibacterial activity against *E. coli* and *S. aureus*, which was further enhanced under blue light irradiation. The hydrogel also functioned as a sensitive strain and motion sensor for detecting body movements, facial expressions, and throat sounds, highlighting its promise for eco-friendly, multifunctional e-skin applications [62]. NajafiKhoshnoo et al. developed a miniaturized, modular, and battery-free wearable pH sensor system fabricated via multimaterial 3D printing of nanomaterials on skin-like flexible substrates, as shown in Figure 4b. The system integrates a disposable polyaniline-based pH sensing module with a reusable NFC-powered communication circuit, enabling real-time, wireless, and noninvasive monitoring without bulky instrumentation. It achieved near-Nernstian sensitivity (51.8 mV pH^−1^), high reproducibility, excellent mechanical flexibility under repeated bending, and strong biocompatibility (>90% cell viability over 7 days). Demonstrated in both artificial sweat and hydrogel-based wound models, this work highlights a scalable strategy for continuous health monitoring and personalized medicine [63].

Moreover, achieving stable skin adhesion without compromising comfort is a crucial challenge, particularly for non-invasive or semi-invasive devices. Techniques such as microneedle arrays, bioinspired dry adhesives, and temperature-sensitive hydrogels offer promising solutions for achieving robust skin-device interfaces with minimal mechanical mismatch [64,65,66]. As shown in Table 1, the successful deployment of wearable sensors in real-world healthcare scenarios depends on a careful balance between material functionality, user comfort, and biological safety. Future research should focus on developing multifunctional materials that combine sensing capability with enhanced biocompatibility and robustness to fully meet the demands of continuous physiological monitoring.

## 3. Sensor Mechanisms and Device Architectures

The performance and functionality of flexible wearable sensors are fundamentally determined by their device architecture and the underlying transduction mechanisms. These sensors are designed to convert various physical, chemical, or physiological stimuli into readable electrical signals with high fidelity, sensitivity, and specificity while maintaining compatibility with soft, conformable form factors required for continuous wear.

In recent years, considerable efforts have been directed toward engineering miniaturized, low-power, and highly integrated sensor platforms that address diverse health monitoring needs. Depending on the nature of the stimulus, wearable sensors can be broadly categorized into mechanical, thermal/electrophysiological, and biochemical types. Each category involves distinct transduction principles and structural design strategies, tailored for real-time and on-body operation. Mechanical sensors are primarily used to detect strain, pressure, and tactile signals, playing an essential role in applications such as gait analysis, pulse detection, and joint movement monitoring. These sensors often rely on piezoresistive, capacitive, piezoelectric, or triboelectric effects to transduce mechanical deformation into electrical outputs. Temperature sensors and bioelectrical signal sensors such as ECG (electrocardiogram) and EMG (electromyogram) are vital for tracking core physiological indicators. These devices require excellent thermal responsiveness or bio-potential sensitivity, often achieved through thermistors, thermoelectric elements, or conductive electrodes in skin-conformal layouts [70,71,72].

In contrast, biochemical sensors enable non-invasive or minimally invasive monitoring of analytes such as glucose, lactate, and electrolytes in bodily fluids (e.g., sweat, interstitial fluid). These sensors typically operate via electrochemical transduction and involve enzymatic or non-enzymatic recognition interfaces integrated with flexible substrates.

### 3.1. Mechanical Sensors

Mechanical sensors are among the most widely studied and applied components in flexible and wearable health monitoring systems. They enable real-time detection of vital biomechanical parameters such as pressure, strain, bending, and tactile interaction, which are critical in applications ranging from pulse and respiration monitoring to gait analysis, joint movement tracking, and tactile perception in human–machine interfaces [67,73,74,75,76,77]. The fundamental working principle of these sensors is based on transducing mechanical deformation into electrical signals. Depending on the transduction mechanism, flexible mechanical sensors are typically classified into the following four types: piezoresistive [78], capacitive [75], piezoelectric [74], and triboelectric [79].

**Figure 5:**
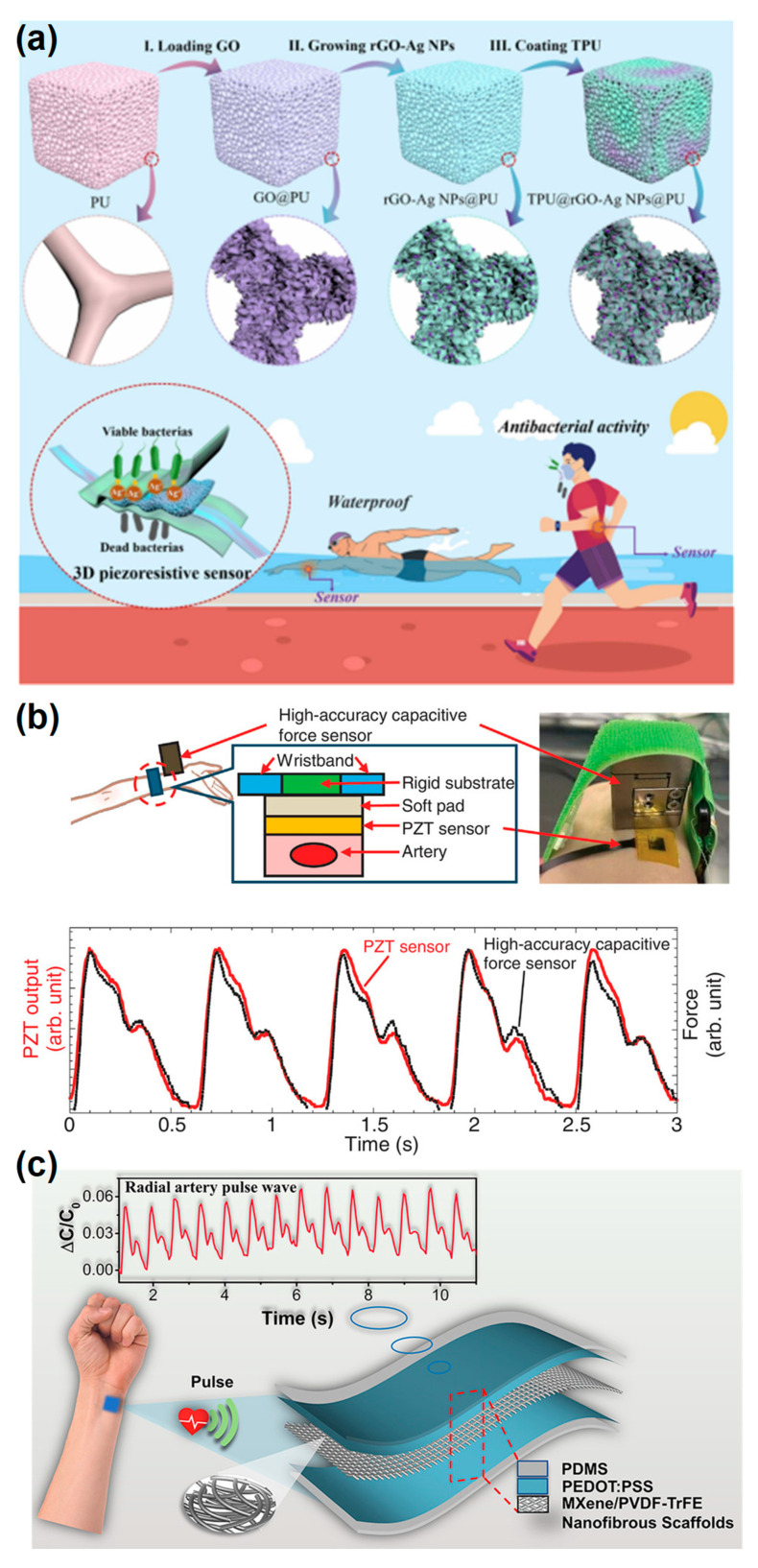
(**a**) Schematic diagram of the preparation and application of 3D wearable multifunctional piezoresistive sensor [34]. (**b**) Schematics illustrating the simultaneous measurement of radial arterial pulse waveforms using our PZT sensor and a high-accuracy capacitive force sensor with a resolution of 0.5 mN [84]. (**c**) Real-time monitoring of the radial artery pulse wave by the proposed wearable capacitive pressure sensor [85]. Reprinted/adapted with permission from [34]. 2023, Elsevier Publishing Ltd. Reprinted/adapted with permission from [85]. 2020, American Chemical Society Publishing Ltd.

Piezoresistive sensors operate by converting mechanical strain into a change in electrical resistance. They are often constructed using conductive nanomaterials such as carbon nanotubes, graphene, or metallic nanowires embedded in elastomeric substrates. The simplicity of their signal readout and high sensitivity to small deformations make piezoresistive sensors well-suited for continuous motion tracking and subtle pulse wave measurements [80]. Recent piezoresistive sensors based on carbon black networks embedded in filter paper has achieved sensitivity of 8.4 kPa^−1^, with fast response time of 95 ms [81]. Capacitive sensors detect changes in capacitance due to variations in the distance or overlapping area between two conductive electrodes separated by a dielectric layer [82]. These sensors are advantageous for their fast response, high stability, and low power consumption. Their flexible architectures can be engineered using patterned microstructures, porous elastomers, or ionic gels to enhance sensitivity and stretchability. Piezoelectric sensors utilize piezoelectric materials such as polyvinylidene fluoride (PVDF) [33,68], zinc oxide (ZnO), or lead zirconate titanate (PZT), which generate electric charges upon mechanical stress. These sensors are particularly effective in detecting dynamic pressures and vibrations, making them suitable for acoustic sensing or motion-related biomedical signals. Triboelectric sensors, based on the triboelectric effect and electrostatic induction, generate electrical outputs from contact and separation of two materials with different triboelectric polarities [83]. They offer a self-powered sensing mechanism and are increasingly integrated into wearable systems for detecting motion, touch, or biomechanical energy. Cao et al. developed a 3D wearable piezoresistive sensor with integrated waterproof and antibacterial properties by incorporating graphene and silver nanoparticles into thermoplastic polyurethane (TPU) and polyurethane (PU) sponges, as shown in Figure 5a. The sensor achieved remarkable sensitivity (GFmax = 152.97 kPa^−1^), fast response times (50 ms/40 ms), and high stability (over 10,000 cycles), making it suitable for a wide range of human activities, from finger movements to subtle body motions like swallowing and breathing. Its waterproof feature ensures reliable operation in humid environments, while the addition of silver nanoparticles provides strong antibacterial activity, making the sensor ideal for long-term, hygienic use [34]. Li et al. developed a wearable PZT piezoelectric sensor for accurate arterial pressure pulse waveform measurement, employing a transfer-free fabrication method for high-quality PZT films directly on flexible substrates, as shown in Figure 5b. The device, mounted with an optimized soft pad, demonstrated exceptional sensitivity and low charge leakage, enabling the accurate measurement of radial arterial pulse waveforms even at low frequencies (1 Hz). With a deep learning model converting sensor signals to blood pressure values without pre-calibration, the sensor achieved a mean absolute error of 5.82 mmHg for systolic and 4.60 mmHg for diastolic pressures [84]. As displayed in Figure 5c, Sharma et al. developed a capacitive pressure sensor based on MXene/poly(vinylidene fluoride-trifluoroethylene) (PVDF-TrFE) composite nanofibrous scaffolds, achieving high sensitivity (0.51 kPa^−1^) and ultra-low detection limits (1.5 Pa). The sensor demonstrated linear sensing over a broad pressure range (0–400 kPa) with excellent durability (>10,000 cycles). The incorporation of MXene increased the dielectric constant by 40%, which significantly enhanced the sensitivity and mechanical stability of the sensor. This work provides a reliable platform for wearable physiological signal monitoring, including pulse, respiration, and muscle movements, while offering potential for next-generation human-machine interfaces [85].

Recent advancements in mechanical sensor design have focused on enhancing stretchability, sensitivity, durability, and spatial resolution. Strategies such as microstructured surface engineering, kirigami-inspired designs, and hybrid material composites have been employed to improve sensor performance under complex, multidimensional strain conditions. Moreover, integration with wireless modules and signal processing units has further expanded the potential of mechanical sensors in real-time health monitoring applications, enabling continuous tracking of body motion, posture, joint health, and even emotional states inferred from subtle mechanical cues.

### 3.2. Temperature and Electrophysiological Sensors

Monitoring physiological parameters such as body temperature and bioelectrical signals including electrocardiogram (ECG) and electromyogram (EMG) is vital for real-time assessment of an individual’s health status, early disease diagnosis, and long-term chronic disease management [24,86,87]. The development of flexible and wearable sensors for these modalities has opened new avenues for continuous, non-invasive, and real-time health monitoring, with applications in both clinical and daily life environments [69,88,89,90,91,92,93]. Flexible ECG and EMG electrodes have achieved skin–electrode impedances below 10 kΩ at 10 Hz, maintaining high signal-to-noise ratios (>20–30 dB) during dynamic motion.

Temperature sensors in wearable formats are typically based on thermoresistive or thermoelectric mechanisms. Thermistors, such as negative temperature coefficient (NTC) materials, exhibit resistance changes with temperature variation and are widely used due to their simplicity and high sensitivity. Thermoelectric sensors, based on the Seebeck effect, can directly convert temperature gradients into voltage, enabling passive and continuous temperature sensing without an external power supply. Materials like polycrystalline silicon, PEDOT:PSS, and metal–semiconductor junctions have been investigated for flexible thermal sensors due to their tunable thermoelectric properties and compatibility with stretchable substrates. For improved skin conformability and minimal thermal lag, recent research has focused on ultrathin sensor architectures using soft polymers such as PDMS and Ecoflex combined with serpentine metal interconnects, nanomesh structures, or liquid metal composites. These innovations allow temperature sensors to adhere intimately to the skin, providing accurate and timely readings during motion or perspiration [94,95].

**Figure 6:**
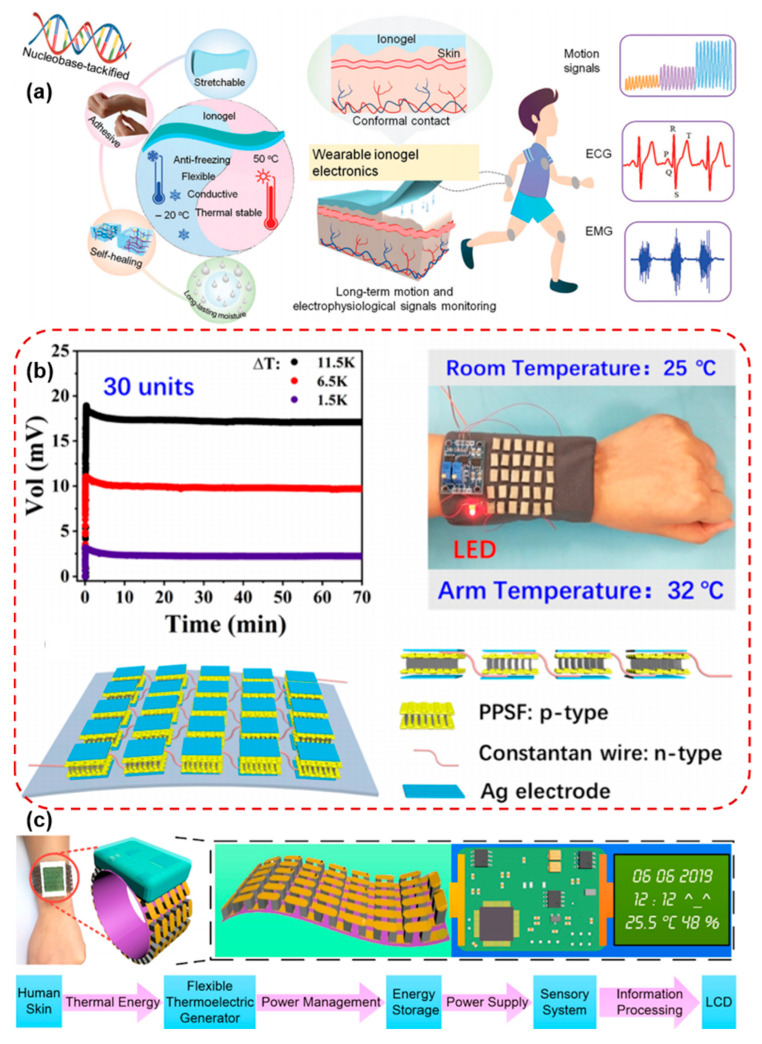
(**a**) Unique characteristics and diverse applications of P(AA-co-AU)-SC-Ga-Al^3+^ ionogels [96]. (**b**) Voltage generated stability of the PPSF thermoelectric devices containing 30 units and LED was lit using the PPSF thermoelectric devices under environmental conditions, ΔT equal to the temperature difference between human skin and the surrounding environment, and construction of the thermoelectric device [97]. (**c**) Schematic view of the self-powered wearable bracelet integrating an f-TEG with a homemade multi-sensory system [98]. Reprinted/adapted with permission from [96]. 2024, John Wiley and Sons Publishing Ltd. Reprinted/adapted with permission from [97]. 2020, American Chemical Society Publishing Ltd. Reprinted/adapted with permission from [98]. 2020, Elsevier Publishing Ltd.

Electrocardiogram (ECG) and electromyogram (EMG) sensors detect the electrical potentials generated by the heart and skeletal muscles, respectively. Traditional gel-based Ag/AgCl electrodes are effective in signal acquisition but suffer from limitations such as skin irritation, dehydration, and reduced performance over extended periods. Flexible dry electrodes have emerged as a promising alternative, offering long-term comfort and stable signal quality. Materials used in flexible electrophysiological electrodes include conductive polymers such as PEDOT:PSS, carbon nanomaterials such as graphene and CNTs and metallic nanowires, which are often embedded in stretchable substrates. These electrodes are designed to maintain low skin electrode impedance, strong adhesion, and high signal-to-noise ratio (SNR), even under dynamic motion. Yan et al. developed a nucleobase-driven wearable ionogel, synthesized via a one-step polymerization process, incorporating gallium (Ga) to enhance both conductivity and mechanical strength as shown in Figure 6a. The ionogel exhibits high stretchability (up to 1600%), excellent self-healing, and environmental resistance, making it ideal for long-term human motion detection and electrophysiological signal monitoring under extreme conditions. The material also demonstrates robust adhesion to human skin, allowing it to function as a bioelectrode for high-quality ECG, EMG, and respiratory signal acquisition [96]. As shown in Figure 6b, Li et al. developed a large-area, wearable self-powered pressure-temperature sensor based on a 3D thermoelectric spacer fabric integrated with PEDOT:PSS, which harvests energy from temperature differences between the body and environment. The sensor demonstrated excellent temperature resolution and fast response for pressure detection across a broad range (200 Pa to 200 kPa). For the first time, a full-body waistcoat-like e-skin was created, capable of independent voltage and current generation under simultaneous temperature and pressure stimuli, marking a significant advancement in multifunctional wearable electronics [97]. Yuan et al. proposed a fully self-powered wearable monitoring system utilizing a flexible thermoelectric generator (f-TEG) optimized through a systematic design process considering power density, material consumption, and power matching for wearable applications, as shown in Figure 6c. The f-TEG, made from bismuth telluride thermoelectric grains on a flexible polyimide substrate, achieves high energy efficiency, with a power density of 3.5 µW/cm^2^ at body temperature. The integration of the f-TEG with a multisensory bracelet allows for the continuous, simultaneous monitoring of human body temperature, humidity, and activity, all powered by body heat, demonstrating a sustainable solution for wearable health monitoring systems [98].

Advanced architectures, such as tattoo-like electronic skin (e-skin), epidermal electronic systems, and textile-integrated sensors, have been demonstrated for real-time ECG/EMG monitoring. These platforms enable unobtrusive tracking of cardiac rhythm, muscle activity, fatigue levels, and even emotional or stress states based on electrophysiological signal patterns. Recent progress also includes integration with wireless transmission modules and AI-based algorithms to enable real-time waveform classification, arrhythmia detection, and muscle fatigue assessment. These innovations are crucial for preventive healthcare, athletic performance monitoring, remote rehabilitation, and early intervention in cardiovascular or neuromuscular disorders.

### 3.3. Biochemical Sensors

Flexible biochemical sensors have emerged as essential tools in wearable health monitoring systems, enabling real-time, non-invasive, and continuous tracking of key biomarkers such as glucose, lactate, electrolytes, cortisol, and alcohol. These sensors provide valuable physiological and metabolic insights beyond traditional physical signals, offering early detection and management of chronic diseases such as diabetes, dehydration, or stress-related conditions. Electrochemical biosensors designed for glucose and lactate monitoring in sweat or interstitial fluid demonstrate detection limits in the micromolar range (1–50 µM), with sensitivities spanning 10–500 µA·mM^−1^·cm^−2^. Biochemical sensors in wearable formats are primarily based on electrochemical transduction mechanisms, including amperometric, potentiometric and conductometric sensing [99]. These modalities translate biochemical interactions at the sensor surface into measurable electrical signals, which are further processed and interpreted.

Electrochemical enzymatic sensors remain the most widely explored configuration, especially for glucose and lactate monitoring. These sensors typically use glucose oxidase [100] or lactate oxidase [101] as biological recognition elements, which catalyze target-specific reactions to generate detectable byproducts such as hydrogen peroxide. The generated current (amperometry) or voltage (potentiometry) correlates directly with analyte concentration. To enhance stability, flexible electrodes are often functionalized with nanomaterials such as gold nanoparticles (AuNPs), carbon nanotubes, or MXene, which increase the electroactive surface area and facilitate electron transfer kinetics.

**Figure 7:**
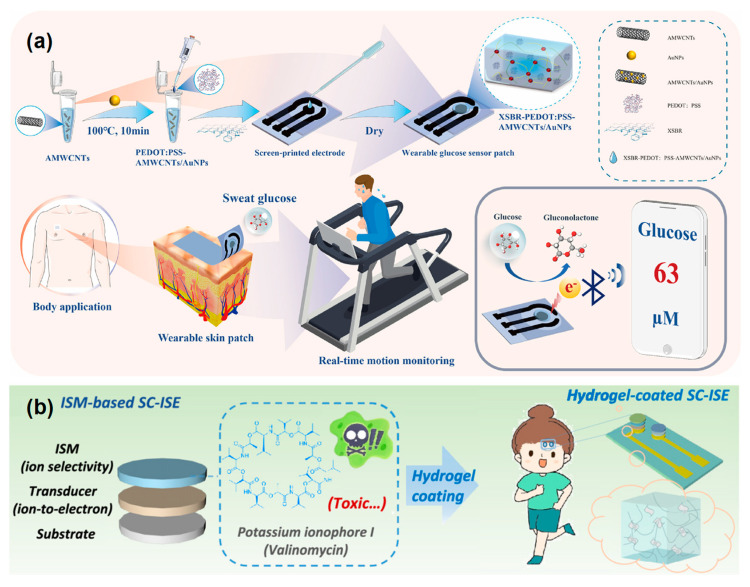
(**a**) Fabrication of the XSBR-PEDOT:PSS-AMWCNTs/AuNPs/SPE and its application as a flexible wearable glucose sensor [104]. (**b**) A typical structure for ISM-based SC-ISE and the proposed GO-PVA hydrogel-coated wearable SC-ISE for sweat K^+^ monitoring [105]. Reprinted/adapted with permission from [104]. 2024, Elsevier Publishing Ltd. Reprinted/adapted with permission from [105]. 2024, American Chemical Society Publishing Ltd.

In recent years, non-enzymatic sensors have gained traction due to their improved operational stability, lower cost, and resistance to environmental fluctuations [102]. These systems utilize materials such as metal oxides, MOFs, and conducting polymers to directly catalyze redox reactions of analytes, eliminating the need for biological elements. This also simplifies fabrication and extends sensor lifespan. For electrolyte sensing detection in sweat or interstitial fluid, ion-selective electrodes (ISEs) are commonly implemented. These sensors incorporate ion-selective membranes embedded with functional ionophores and plasticizers, providing high specificity and sensitivity. Wearable ISEs integrated into skin patches, microneedles, or textile substrates have shown reliable performance in real-time hydration tracking, athletic performance monitoring, and electrolyte imbalance warning systems [103]. Chen et al. developed a flexible non-enzymatic electrochemical sensor for sweat glucose monitoring by integrating gold nanoparticles on aminated multi-walled carbon nanotubes with XSBR and PEDOT:PSS as shown in Figure 7a. The composite material provided high conductivity, mechanical flexibility, and strong catalytic activity under neutral conditions, enabling sensitive detection with a low limit of 3.2 μM. The wearable device demonstrated excellent stability against bending, strong selectivity against common interferents, and reliable correlation with blood glucose levels in real human sweat samples [104]. Liu et al. developed a graphene oxide–poly(vinyl alcohol) (GO-PVA) hydrogel coating for solid-contact ion-selective electrodes, as shown in Figure 7b. The hydrogel provided enhanced mechanical robustness and biocompatibility while preserving Nernstian sensitivity, high selectivity, and strong anti-interference capability. Flexible electrodes fabricated on PET substrates demonstrated stable performance under bending and were successfully integrated with solid-contact reference electrodes. Importantly, the wearable device enabled accurate real-time monitoring of sweat potassium, validated against ion chromatography [105].

The integration of biochemical sensors with flexible substrates, such as PDMS, Ecoflex, or hydrogel matrices, ensures skin compliance and mechanical robustness during movement. To prevent biofouling and signal drift, surface modification strategies such as antifouling coatings and encapsulation layers are often employed, enhancing biocompatibility and long-term functionality.

Moreover, combining biochemical sensing with real-time wireless data transmission and machine learning analytics further enhances the practicality of wearable biochemical monitoring systems. For instance, multianalyte sensor arrays with data fusion algorithms enable comprehensive health assessments, accounting for physiological correlations among glucose, lactate, pH, and temperature. Looking forward, advances in sweat-based diagnostics, non-invasive microneedle sensors, and biofuel-powered biosensors are expected to transform flexible biochemical sensors into indispensable tools for personalized healthcare, sports science, and continuous disease management.

## 4. System-Level Sensor Integration and Applications

The growing demand for continuous, real-time, and personalized healthcare has significantly accelerated the development and deployment of flexible and wearable sensor technologies. These systems serve as non-invasive tools for capturing dynamic physiological signals directly from the human body, enabling the transition from reactive to proactive healthcare models. By providing timely and longitudinal data outside of clinical environments, wearable sensors offer a powerful platform for early diagnosis, disease prevention, and long-term health management.

Real-time health monitoring involves the detection and interpretation of vital physiological, biomechanical, and biochemical signals. The ability of wearable sensors to conform to the body’s surface allows for unobtrusive integration with daily activities, making them suitable for both clinical and consumer-level applications. Through advanced material engineering, microfabrication, and low-power electronics, modern wearable systems achieve high sensitivity, mechanical compliance, and wireless connectivity, meeting the critical requirements for continuous operation in ambulatory settings. In recent years, flexible sensors have been integrated into patches, textiles, wristbands, and epidermal electronics to monitor vital signs, such as heart rate, body temperature, respiratory rate, and blood oxygen saturation. Simultaneously, sensors capable of motion and activity tracking such as accelerometers, strain sensors, and gyroscopes have enabled functional assessments in rehabilitation, posture monitoring, and gait analysis, as shown in Table 2. Moreover, emerging biochemical and multimodal sensors are facilitating the management of chronic diseases, including diabetes, cardiovascular disorders, and sleep apnea, by detecting biomarkers or physiological anomalies in real time. These application areas are supported by robust signal acquisition, on-device processing, and wireless communication systems, often enhanced by machine learning algorithms for pattern recognition and anomaly detection.

### 4.1. Vital Signs Monitoring

Vital signs, including heart rate, body temperature, respiratory rate, and blood oxygen saturation, are fundamental physiological parameters that provide essential information about an individual’s health status. Continuous and real-time monitoring of these signals using flexible and wearable sensors enables early detection of abnormalities, facilitates personalized medical interventions, and supports chronic disease management outside of hospital environments. Flexible heart rate sensors typically employ either photoplethysmography (PPG) or electrocardiography (ECG) principles [108]. PPG-based systems utilize light-emitting diodes and photodetectors embedded in flexible substrates to detect changes in blood volume, whereas ECG sensors rely on conformal electrodes that adhere to the skin to capture bioelectric signals generated by cardiac activity. Recent advances in conductive hydrogels, stretchable electrodes, and textile-based sensors have significantly improved signal quality and long-term wearability [13,81,109].

**Figure 8:**
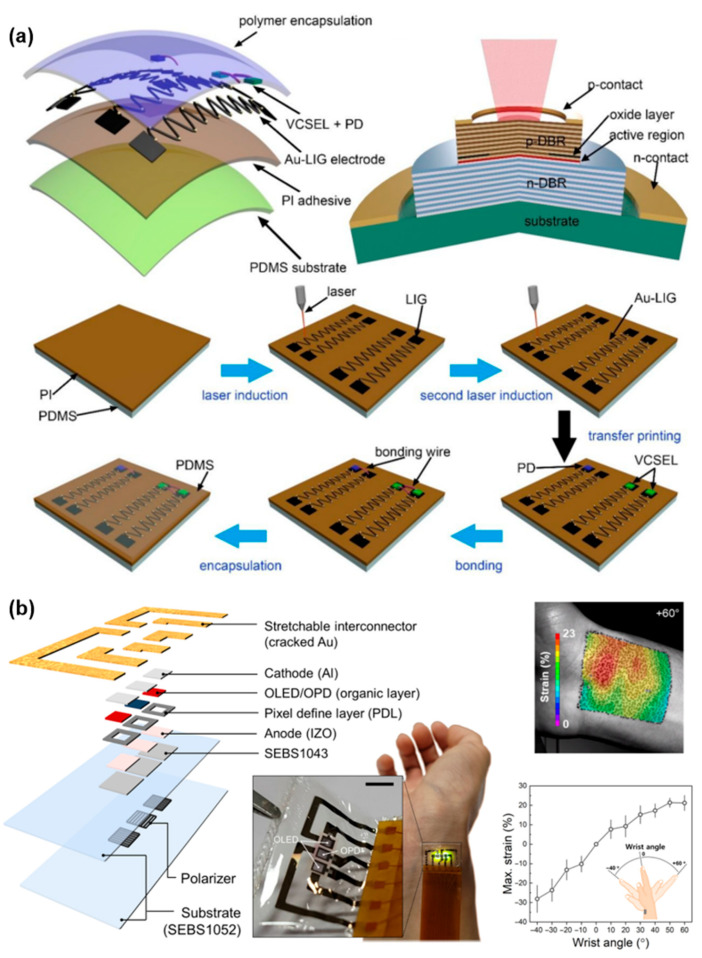
(**a**) Schematic illustration of the wearable SpO2 monitoring system based on a VCSEL and Si PD on a flexible PDMS substrate and the structure of VCSEL and fabrication process of a wearable SpO2 device [110]. (**b**) Schematic of the layer layout of the OPA sensor and stretchable patch-type sensor in contact with the skin on the inner side of the wrist (scale bar, 3 mm). Measured strain distribution on the skin on the inner side of the wrist at a wrist angle of +60°. The time-dependent surface profile of the skin recorded with a motion capture system using digital image correlation (DIC) analyses. Maximum strain on the skin with respect to the wrist angle in the range of −40° (flexion) to +60° (extension) [111]. Reprinted/adapted with permission from [110]. 2022, American Chemical Society Publishing Ltd.

Body temperature monitoring is commonly achieved using resistive temperature detectors (RTDs), thermistors, or thermoelectric materials integrated into soft, skin-mountable platforms. These sensors enable continuous thermoregulation assessment and fever detection, which are particularly important for infectious disease screening and patient monitoring in critical care. Respiratory rate monitoring can be accomplished by measuring thoracic or abdominal motion through strain sensors, or by detecting humidity and temperature variations in exhaled breath using flexible humidity sensors. Stretchable piezoresistive and capacitive sensors adhered to the chest region have shown promise for capturing the mechanical dynamics of respiration with high fidelity. Blood oxygen saturation is another vital metric, typically measured through reflectance or transmissive pulse oximetry. Wearable oximeters that integrate flexible optoelectronic components and signal processing circuits have been developed to continuously monitor blood oxygen saturation levels, particularly beneficial for patients with chronic pulmonary conditions or during sleep studies. Li et al. demonstrated a wearable and biocompatible blood oxygen sensor by heterogeneously integrating narrowband vertical cavity surface emitting lasers and a thinned Si photodetector onto a laser-induced graphene–gold hybrid flexible electrode, as shown in Figure 8a. The Au-LIG electrode fabrication enabled low-cost, scalable, and highly flexible substrates, while the integrated surface emitting lasers provided narrow emission bandwidths (1.8 and 0.8 nm at 680 and 808 nm) that enhanced photoplethysmography sensitivity [110]. In Figure 8b, Lee et al. introduced a stretchable, skin-conformable PPG sensor that incorporates an orthogonal polarizer–analyzer structure to effectively suppress motion artifacts. By selectively filtering out light scattered from superficial skin layers while retaining signals from deeper blood vessels, the device reduced motion noise by more than tenfold compared with rigid sensors under large wrist-angle movements. The integration of organic photodiodes and microcracked gold interconnects on elastomer substrates enabled both mechanical stretchability and a high signal-to-noise ratio [111].

The integration of these sensor types into wearable systems requires careful consideration of comfort, accuracy, power consumption, and data transmission protocols. In recent years, many commercial and experimental systems have demonstrated the feasibility of real-time vital signs monitoring in daily life, paving the way for personalized and preventive healthcare paradigms.

### 4.2. Motion and Activity Tracking

Monitoring human motion and physical activity plays a crucial role in applications such as rehabilitation assessment, gait analysis, posture recognition, and athletic performance optimization [112]. Flexible and wearable motion sensors offer an unobtrusive and continuous means to evaluate body kinematics in both clinical and daily-life scenarios, enabling personalized feedback and data-driven interventions. Strain and pressure sensors are commonly employed to capture mechanical deformations associated with body movement. These sensors are typically fabricated using piezoresistive, capacitive, or piezoelectric mechanisms and are integrated into textiles, patches, or stretchable substrates that conform to joints and muscles. For instance, soft strain sensors mounted on knees or elbows can quantify joint angles and movement frequency, providing real-time input for physical therapy monitoring or movement disorder diagnosis. When combined with machine learning algorithms, these sensors can be used to classify complex motion patterns, such as walking, running, or falling. Recent studies have demonstrated the use of multi-sensor arrays placed on different body parts to achieve full-body motion capture without relying on external cameras or bulky equipment.

**Figure 9:**
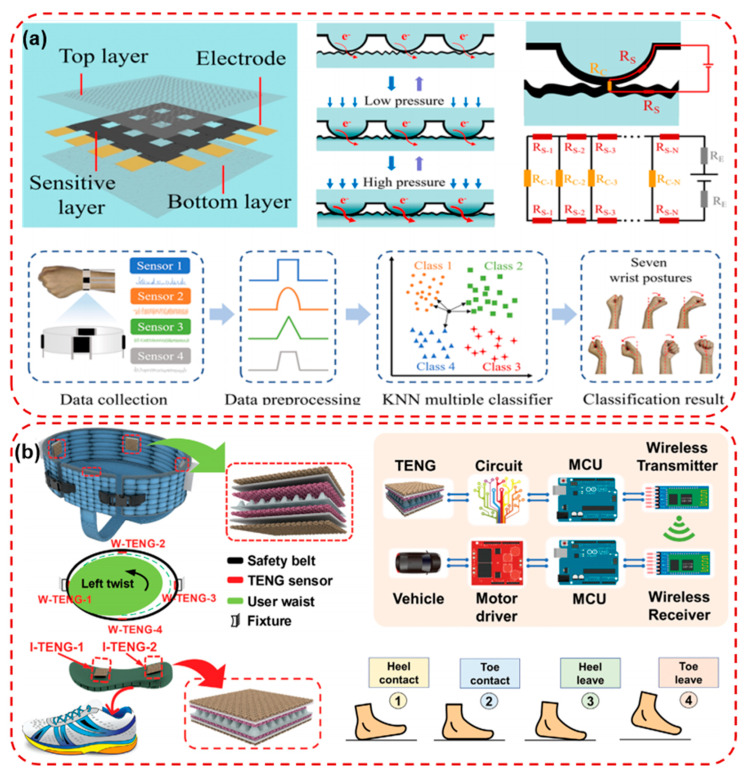
(**a**) Sensing mechanism of the PDMS film-based PEDOT:PSS/CNC flexible pressure sensor and schematic diagram of wrist attitude recognition process based on the KNN algorithm [115]. (**b**) Schematics and working mechanism of the intelligent safety belt with four TENG sensors. The safety belt is fixed to the fixture similar to the rigid bracket of the rehabilitation robot and four states of a typical gait analysis contact cycle [107]. Reprinted/adapted with permission from [115]. 2024, American Chemical Society Publishing Ltd.

Posture recognition and correction represent another important area, particularly for individuals with spinal disorders or those undergoing rehabilitation. Flexible posture sensors can monitor spinal curvature and detect prolonged periods of improper posture, triggering alerts or feedback signals to encourage corrective behavior. These devices are often integrated into smart garments or adhesive patches for unobtrusive use during daily activities [32,113,114]. Zhang et al. designed a PDMS film-based flexible pressure sensor array with surface protruding structures, combined with a PEDOT:PSS/cellulose nanocrystal conductive layer, fabricated through a simple and low-cost template transfer method as shown in Figure 9a. The device achieved high sensitivity (2.32 kPa^−1^), a wide detection range (0–100 kPa), fast response (240 ms), and excellent durability over 2000 cycles. Beyond detecting macro and subtle human motions such as finger bending, pulse, and respiration, the 4 × 4 sensor array enabled pressure mapping and object shape recognition. Furthermore, a smart wristband integrated with four sensors, coupled with a KNN algorithm, achieved 99.52% accuracy in wrist posture classification [115]. Zhang et al. also developed a textile-based triboelectric sensory system for gait analysis and waist motion capture, integrating pyramid-patterned triboelectric layers encapsulated in TPU-coated fabric to ensure stability against humidity and long-term wear as shown in Figure 9b. The system consisted of a smart insole and an intelligent safety belt, both capable of generating self-powered signals for motion monitoring, user recognition, and rehabilitation training. By coupling triboelectric sensors with machine learning algorithms, the device achieved 98.4% accuracy in distinguishing different users and enabled personalized rehabilitation plans while preserving privacy. Furthermore, integration with a lower-limb rehabilitation robot and gaming-enhanced training demonstrated its potential for immersive, IoT-based smart healthcare applications [107].

Moreover, real-time motion tracking systems can be synchronized with mobile devices or cloud platforms to enable remote monitoring by healthcare providers [116]. This not only facilitates telemedicine and home-based rehabilitation but also supports long-term activity trend analysis for health risk prediction and behavioral modification. The integration of motion and activity tracking capabilities into wearable systems enhances their value beyond basic health monitoring, offering new possibilities for personalized medicine, sports science, and assistive technologies.

### 4.3. Chronic Disease and Rehabilitation

Chronic diseases such as diabetes, cardiovascular disorders, and respiratory illnesses remain leading causes of morbidity and mortality worldwide. Effective management and rehabilitation of these conditions require continuous, non-invasive monitoring of relevant physiological parameters, an area where flexible and wearable sensors offer transformative potential. In the context of diabetes management, wearable biochemical sensors capable of detecting glucose levels through sweat, interstitial fluid, or tear fluid have emerged as promising alternatives to traditional blood-based testing. These sensors utilize electrochemical or enzymatic detection mechanisms and can be integrated into skin patches or contact lenses. Their continuous operation enables real-time glycemic profiling, early detection of hypoglycemic episodes, and data-driven insulin dosing adjustments.

For cardiovascular diseases, flexible ECG sensors that conform to the skin surface enable high-fidelity monitoring of heart rate and rhythm abnormalities such as arrhythmias. These devices offer enhanced comfort and longer wearability compared to conventional rigid electrodes, making them ideal for ambulatory or home-based cardiac assessments. Additionally, pulse oximeters and arterial stiffness monitors are being integrated into flexible platforms for continuous tracking of blood oxygen saturation and vascular health, which are critical indicators in hypertension and heart failure management [13,117,118].

**Figure 10:**
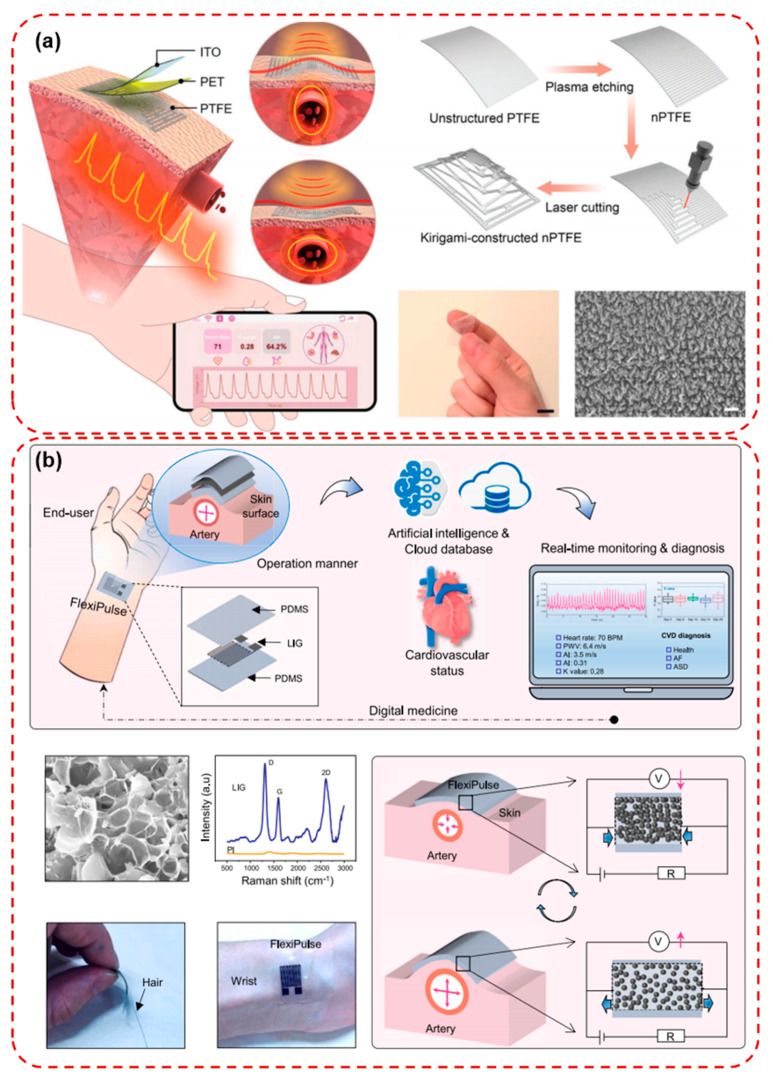
(**a**) Schematic illustration of the as-fabricated KIP sensor containing a three-layer configuration: PTFE film and PET substrate as the two electrification layers, with a third layer of ITO coated onto the surface of PET acting as electrode. Schematic illustration showing the fabrication procedure for kirigami constructed nanowire PTFE. SEM images of nanowire-patterned PTFE (scale bar: 1 µm). Photograph of the as-fabricated KIP sensor, demonstrating its excellent flexibility (scale bar: 1 cm) and a KIP sensor-based wireless cardiovascular monitoring system performs continuous pulse wave monitoring, and cardiovascular parameters are simultaneously displayed on a mobile phone via wireless transmission [123]. (**b**) Schematic illustration presents FlexiPulse for real-time cardiovascular monitoring. SEM images present micromorphological porous structures of laser-induced graphene (LIG). Scale bar: 5 mm. Fabricated FlexiPulse compared with hair and self-attaching on wrist skin [124]. Reprinted/adapted with permission from [123]. 2022, John Wiley and Sons Publishing Ltd. Reprinted/adapted with permission from [124]. 2023, Elsevier Publishing Ltd.

In the area of respiratory health, wearable systems capable of monitoring respiratory rate, airflow patterns, and thoracic movement are being deployed for conditions such as sleep apnea, asthma, and chronic obstructive pulmonary disease. These sensors are often embedded in chest bands or adhesive patches and can detect irregular breathing events, supporting early intervention and personalized therapy adjustments. Rehabilitation following injury, surgery, or stroke also benefits significantly from wearable sensing technologies. Devices designed to monitor joint motion, muscle activation via EMG, and gait patterns enable clinicians to quantitatively assess recovery progress and tailor rehabilitation protocols accordingly. Furthermore, real-time feedback mechanisms embedded within these systems can guide patients through physical therapy exercises, improve compliance, and reduce the need for in-person supervision [119,120,121,122]. Meng et al. developed a kirigami-inspired pressure (KIP) sensor that combines nanowire-patterned PTFE and PET layers to achieve high conformability and motion artifact resistance in wearable cardiovascular monitoring as shown in Figure 10a. The unique vertical kirigami structure enabled large elastic deformation, resulting in superior sensitivity (35.2 mV Pa^−1^), a wide frequency response, and excellent durability over 84,000 cycles. Integrated into a wireless system, the sensor provided real-time, accurate pulse wave monitoring across various arterial sites, even under prestressing pressures and body movements [123]. In Figure 10b, Ma et al. developed FlexiPulse, a low-cost, flexible, and conformally skin-integrated pulse sensor fabricated by an eco-friendly laser direct-engraving process of porous graphene. The ultrathin sensor exhibited high sensitivity, durability over 24,000 cycles, and reliable monitoring of multiple arterial sites without requiring external pressure. Clinically validated, FlexiPulse achieved >93% accuracy in detecting subtle cardiovascular changes and, when combined with machine learning, classified cardiovascular disease events such as atrial fibrillation and atrial septal defect with an accuracy of 98.7% [124].

Overall, the integration of flexible and wearable sensors into chronic disease management and rehabilitation frameworks provides a pathway toward continuous, individualized healthcare, aligning with the paradigm of preventive and precision medicine.

Practical deployment of flexible wearable sensors requires careful consideration of power management, clinical validation, and data security. Low-power circuit design and energy-harvesting strategies (BLE modules consuming <1 mW) enable continuous, long-term operation without frequent recharging [125,126,127]. At the same time, compliance with clinical standards such as glucose sensors achieving ±15% accuracy in 95% of cases (ISO 15197) [128] or wearable ECG sensors with heart rate errors below ±2 bpm ensures reliability and regulatory acceptance. Together, these considerations highlight that beyond materials and sensing principles, practical integration into healthcare systems must address energy efficiency, clinical accuracy, and data security to ensure widespread adoption [129,130].

## 5. Conclusions

Flexible and wearable sensors for real-time health monitoring represent a rapidly advancing and interdisciplinary field, integrating innovations in materials science, device engineering, system integration, and data analytics. These sensors offer unique advantages such as mechanical flexibility, biocompatibility, and conformability to the human body, enabling continuous, non-invasive, and unobtrusive monitoring of vital physiological and biochemical signals. This review has systematically discussed the recent progress across the entire development chain from substrate and functional materials to device architectures and system-level integration highlighting the significance of novel sensing principles and intelligent electronics. Additionally, various real-world healthcare applications were explored, including vital signs monitoring, motion tracking, chronic disease management, and rehabilitation support, all of which demonstrate the transformative potential of these technologies in promoting personalized and preventive medicine.

Despite the remarkable progress, several challenges remain before flexible wearable sensors can be widely adopted in clinical and consumer healthcare settings. First, material-related challenges persist in achieving long-term biocompatibility and mechanical robustness under dynamic physiological conditions. For instance, developing stretchable yet durable substrates and encapsulation strategies is essential for maintaining signal stability and user comfort over extended use. Second, sensor accuracy and reliability remain critical issues, particularly in harsh and variable environments. Minimizing signal drift, motion artifacts, and cross-sensitivity to environmental stimuli is necessary to ensure precise and consistent readings.

Third, the integration of multiple sensing modalities—mechanical, thermal, electrophysiological, and biochemical—into a single platform poses design and fabrication complexities. Achieving seamless multi-modal signal acquisition without compromising device compactness or flexibility is a key future goal. Fourth, power consumption and energy management remain limiting factors for long-term operation. Future research should emphasize the development of ultra-low-power electronics, energy harvesting strategies, and efficient wireless data transmission systems. Fifth, on the system level, secure data processing, storage, and real-time analytics via AI/ML algorithms raise challenges related to computational efficiency, interpretability, and user privacy. Developing edge computing frameworks with on-device intelligence will be crucial for real-time decision making.

In future research, a holistic design approach combining material innovation, device miniaturization, intelligent algorithms, and human-centered interfaces will be necessary. Ultimately, such efforts will accelerate the development of next-generation flexible sensing systems that are not only high-performance and user-friendly but also capable of fundamentally reshaping modern healthcare delivery.

## Figures and Tables

**Table 1:** Summary of commonly used materials for flexible and wearable sensors.

Category	Representative Materials	Key Properties	Bio-Compatibility/Stability	Ref
Substrate Materials	Polyimide, PDMS, Ecoflex, Hydrogel, Textile, Paper	Flexibility, stretchability, lightweight, gas permeability	Thermally stable, not biodegradable, skin-friendly, long-term adhesion, good biocompatibility but dehydration issue	[14,30,40,45]
Conductive Polymers	PEDOT:PSS, Polyaniline, Polypyrrole	High conductivity, tunable mechanical properties, solution processability	Generally biocompatible, widely used for skin-contact electrodes	[23,65]
Carbon-Based Materials	Graphene, CNTs	High electrical conductivity, high surface area, mechanical robustness	Good skin compatibility, biocompatibility depends on functionalization	[47,67]
2D Materials	Ti_3_C_2_Tx MXene and Derivatives	Metallic conductivity, hydrophilicity, high sensitivity	Surface prone to oxidation, stability improved by encapsulation	[54,56]
Metal Nanostructures	Ag Nanowires, Au Nanoparticles	High conductivity, transparency, stretchability (Ag NW networks)	AuNPs biocompatible	[52,55]
Functional Inorganic Materials	PVDF, ZnO, Bi_2_Te_3_, Metal Oxides (NiO, CuO)	Energy harvesting, pressure/temperature sensitivity	Less biocompatible, need encapsulation	[67,68]
Composite Materials	Polymer–Nanomaterial Hybrids	Synergistic flexibility, conductivity, high sensitivity	Biocompatibility depends on polymer matrix, composites enhance stability	[63,69]

**Table 2:** Summary of recent flexible wearable sensors for health monitoring.

Sensing Mechanism	Representative Materials/Structures	Figures of Merit	Application Domains	Representative Works
Piezoresistive	CNTs, graphene films, PEDOT:PSS composites, textile-integrated strain gauges	High sensitivity, fast response, simple readout	Motion tracking, gait analysis, pulse monitoring	[78,80,81]
Capacitive	Microstructured elastomers (PDMS, Ecoflex), Ag NWs electrodes	High linearity, low hysteresis, stable under deformation	Pressure mapping, respiratory monitoring, tactile sensing	[25,75,90]
Piezoelectric	PVDF, ZnO nanowires, PZT thin films	Self-powered sensing, high dynamic response	Pulse wave monitoring, voice recognition, joint motion detection	[74,77,84]
Triboelectric	Micro/nanopatterned polymers, textile-integrated TENGs	Energy harvesting + sensing, broad material choices	Motion tracking, energy-autonomous sensors	[79,106,107]
Thermoresistive	Metal thin films, PEDOT:PSS, Bi_2_Te_3_-based composites	Temperature resolution < 0.1 °C, passive operation	Body temperature monitoring, fever screening	[23,88,91]
Electrochemical	MXenes, metal oxides (Ni, Cu, Co-based)	High stability, enzyme-free catalysis	Glucose, alcohol, electrolyte detection	[39,54,104]
Bioelectrical (ECG/EMG)	Graphene electrodes, CNT yarns, PEDOT:PSS hydrogel	Low impedance (<10 kΩ), SNR > 20 dB	ECG, EMG, human–machine interface, rehabilitation	[12,17,25]

## Data Availability

No new data were created or analyzed in this study.
